# The effect of time of onset on community preferences for health states: an exploratory study

**DOI:** 10.1186/1477-7525-9-6

**Published:** 2011-01-20

**Authors:** Eve Wittenberg

**Affiliations:** 1 Heller School for Social Policy and Management, Brandeis University, Waltham, MA

## Abstract

**Background:**

Health state descriptions used to describe hypothetical scenarios in community-perspective utility surveys commonly omit detail on the time of onset of a condition, despite our knowledge that among patients who have a condition, experience affects the value assigned to that condition. The debate regarding whose values to use in cost utility analysis is based in part on this observed difference between values depending on the perspective from which they are measured. This research explores the effect on community preferences for hypothetical health states of including the time of onset of a health condition in the health state description, to investigate whether this information induces community respondents to provide values closer to those of patients with experience with a condition. The goal of the research is to bridge the gap between patient and community preferences.

**Methods:**

A survey of community-perspective preferences for hypothetical health states was conducted among a convenience sample of healthy adults recruited from a hospital consortium's research volunteer pool. Standard gambles for three hypothetical health states of varying severity were compared across three frames describing time of onset: six months prior onset, current onset, and no onset specified in the description. Results were compared within health state across times of onset, controlling for respondent characteristics known to affect utility scores. Sub-analyses were conducted to confirm results on values meeting inclusion criteria indicating a minimum level of understanding and compliance with the valuation task.

**Results:**

Standard gamble scores from 368 completed surveys were not significantly different across times of onset described in the health state descriptions regardless of health condition severity and controlling for respondent characteristics. Similar results were found in the subset of 292 responses that excluded illogical and invariant responses.

**Conclusions:**

The inclusion of information on the time of onset of a health condition in community-perspective utility survey health state descriptions may not be salient to or may not induce expression of preferences related to disease onset among respondents. Further research is required to understand community preferences regarding condition onset, and how such information might be integrated into health state descriptions to optimize the validity of utility data. Improved understanding of how the design and presentation of health state descriptions affect responses will be useful to eliciting valid preferences for incorporation into decision making.

## Background

As demands to improve efficiency of health care expenditures increase, valid and accurate measures of the effectiveness of health interventions are becoming increasingly important[1]. Primary among such measures are health utilities, the basis for quality adjusted life years (QALYs)[2]. Methods of measuring health utilities have been evolving since they were originally proposed by von Neumann and Morgenstern[3], with improvements, refinements and adaptations occupying investigators from psychology to economics[4]. This paper addresses one specific aspect of utility elicitation, the time of onset of illness, and how its inclusion in health state descriptions developed specifically for the elicitation of community perspective preferences affects the articulation of those preferences. The goal of the study was to illuminate utility survey design elements underlying well-documented differences between patient and community-perspective values.

A health state may be defined as an event that begins with an occurrence, sometimes develops and changes over time, and usually has a resolution, including death. Acute states have a short time span from beginning to end while chronic states take many turns over long duration from start to finish. Quality adjusted life years incorporate the duration of each phase of an illness into a calculation that results in the overall value of the course of disease, including changes in severity and quality of life over time. A specific health state occurring at one point in time during the course of an illness or health condition is valued through the utility assigned to that state, and duration is incorporated into the QALY calculation through a multiplication of time (duration) and utility.

It may be, however, that individuals' utility for a certain state depends both on when that state began and how long it persists (as well as what preceded and follows it). When it began, or time of onset, may determine the level of adaptation that the individual is experiencing at the point in time that the health state is occurring, with greater time since onset often indicating greater adaptation to a state and hence higher utility[5,6]. In addition, it may be that the transition from healthy to ill, meaning the time surrounding the onset of a disease or condition, infers a transition process that has an altogether different utility value from that assigned to a state once it has been underway for some period of time. Hence health states of recent occurrence may include this transition factor in their utility while those of longer time since inception may not. States of longer duration may instead include emotional elements associated with the passage of time, including hope, despair, and inference of prognosis. In all, the time of onset of an illness or condition may affect the utility assigned to a particular state separate from the time-independent assessment of the state.

Experienced utilities, meaning those elicited from persons who have a particular condition (i.e., "patient-perspective" utilities) likely incorporate these and perhaps other elements of value in the scores assigned to them. Community-perspective utilities do not benefit from experience with a state, and therefore rely on the information provided in descriptions used in the elicitation process to convey all aspects of value related to a condition[6,7]. Time since onset is generally not included in the health state descriptions used in community-perspective utility surveys, suggesting a potential bias of omission.

In the elicitation of community-perspective utilities, those preferred for cost-effectiveness analysis[8], the question arises of whether these elements that accompany the patient-perspective are salient or can be incorporated into elicited values, or both, and by what mechanism this can be achieved. This paper addresses the specific question of how the statement of disease onset affects utility values for hypothetical states evaluated by community members: whether the general practice of omitting this information from health state descriptions biases utility scores by omitting details that would otherwise be informative to community-perspective evaluations. To an inexperienced (i.e., community) evaluator, the time of onset of a condition may imply adaptation to disease, the fear of transition to disease, or the dread and hopelessness that accompanies long-term illness. While descriptors used in community-perspective valuations that increase the accuracy of health state descriptions are desirable, time of onset is not usually mentioned in utility surveys. This study attempted to integrate information on the experience with a condition into hypothetical health state descriptions in order to allow community-perspective respondents to use this information in their valuations. We hypothesized that the inclusion of time of onset information in community-perspective surveys would allow respondents to incorporate coping, adjustment, and affective components of fear, hope and dread into their valuations and therefore more closely parallel an experienced (patient) perspective. Our goal was to inform the design of utility surveys and the interpretation of results.

## Methods

### Design

**Figure 1:**
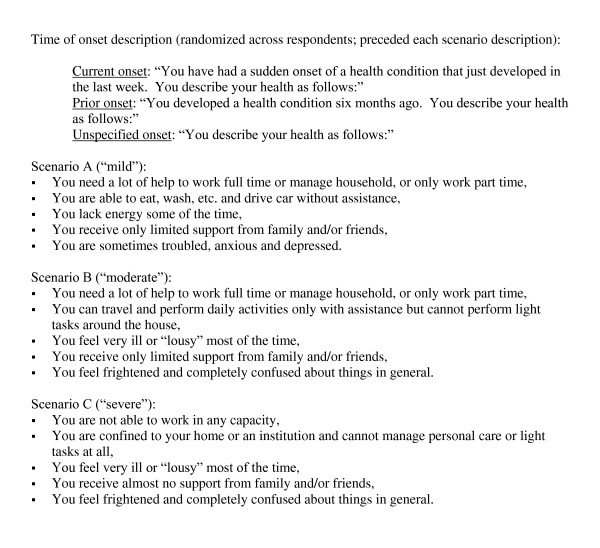
**Health state scenario descriptions**.

We conducted a cross-sectional utility survey of community members for hypothetical health states with a three-part split sample by time of onset of the conditions. Each respondent valued the same three hypothetical health states using the standard gamble, with their randomly assigned onset frame. The three states described different levels of disability, including mild, moderate and severe, in terms of a generic, unspecified disease described using the format of the Quality of Life Index (five dimensions of health (ability to work, self care, energy level, social support, anxiety/depression), each of which is described in one of three levels of severity[9]; Figure 1). The three randomly-assigned onset frames were described as follows: one-third were told that each of the three health states commenced six months prior ("prior onset"), one-third were told they began one week ago ("current onset"), and one-third were presented with the descriptions with no additional information about their time of onset ("unspecified onset").

The survey was administered over the internet, with recruited participants directed to the web site and all answers provided anonymously. The standard gamble (SG) was presented in iterative form using a bisection pattern with endpoints of dead and perfect health. Both numerical probabilities and visual aids were presented for the gamble, and up to two repeats of the SG response were permitted and the final answer was used for analyses. The study was approved by the Institutional Review Board of Partners Healthcare System.

### Sample

A community sample was approximated by employing a sampling frame developed from a pre-existing volunteer pool of individuals recruited for clinical research by a major hospital consortium in the Boston, MA area. Names and either electronic or postal mail addresses of individuals who self-identified as "healthy volunteers" were maintained by the hospital, and recruitment messages were sent by the respondent's preferred method of contact. Recruitment was conducted by a hospital intermediary to maintain participant anonymity, and information on undelivered mail was not provided to the investigator. Respondents were invited to visit a website for the survey only once to minimize respondent recruitment burden. The study was designed to recruit 40 respondents per time of onset group, or 120 respondents in total, which would provide 80% power to detect differences in mean utility scores between groups of 0.13, based on 5% significance and an expected standard deviation in mean utility score of 0.2. Utility scores are highly variable and a difference of 0.15 or more between groups would be considered a meaningful difference[10]. In fact, recruitment exceeded expectations and the resulting sample was far larger, resulting in greater power to detect differences between groups.

### Analysis

The analysis focused on identifying any potential effect of time of onset on community values for the health states. Both the entire survey sample and a subset of individuals who met criteria indicating a minimum level of understanding and compliance with the valuation task were used for analysis. Descriptive statistics were calculated to characterize the sample and the utility scores provided for the three different hypothetical health states. Regression models were built to test two hypotheses regarding the effect of time of onset on community-perspective SG scores for hypothetical states: (1) that prior onset conditions would be valued higher than current onset conditions, and (2) that the inclusion of a specified onset in the description, either current or prior, would be valued differently than no information regarding onset (i.e., unspecified onset).

A subset analysis based on response criteria was conducted to explore the stability of the main analysis results when potentially questionable survey results were excluded. The exclusion of illogical and "non-trader" (i.e., invariant) responses from utility surveys has been debated in the field, with some suggesting that omission increases the validity of results[11-13]. We therefore conducted our analyses including and excluding these responses to provide confirmation of our results. Our inclusion criteria were logic and variance: logical responses were those in which the SG value for the mild state was greater than that for the moderate state, which was greater than that for the severe state. Illogical responses violate this ordering and suggest miscomprehension of the valuation task or confusion. Responses demonstrating variance were those in which at least one SG score was different than others, in contrast to invariant responses in which the same score is given for every state. Such responses are often considered "protest" responses in which the respondent is averse to the premise of the valuation task and therefore refuses to trade any risk of death for improved health, or are expressions of extreme risk aversion or a lack of sensitivity of the instrument[11,14,15]. Both illogical and invariant responses may introduce noise or bias into results.

Generalized linear modeling was used to analyze the entire sample and the logical/variant subsample. A model was built for each of the three health states: the dependent variable was the SG score and the main independent variable was the time of onset frame. Time of onset was coded as three dummy variables, "unspecified onset," "prior onset" and "current onset," with prior as the reference group to test the hypothesis that prior > current and unspecified as the reference group to test the hypothesis that unspecified ≠ current or prior. Covariates believed *a priori *to affect valuations were included in the models as control variables, including age (continuous), education (college or higher education versus less), gender (female versus male), race (white versus all other), health status (categorical with 1 = excellent and higher values = worse health status), religiosity (identify as religious versus do not), and dependent children (children < 18 years in household versus not). Statistical significance was assessed with two-sided tests and p-values of 0.05. Analyses were conducted using SAS version 9.2 (SAS Institute, Cary, NC).

## Results

A total of 8,380 volunteer names were identified in the hospital database and used for recruitment. Six hundred and twenty-one visits to the web site resulted in 368 complete responses, of which 292 met logic and variance criteria for inclusion in the subset analysis. Respondents were primarily female (76%), white (88%), and well-educated (72% completed college or higher education), with a mean age of 40 years (Table 1). Compared with the US population, the study sample contained more women, more white and fewer black individuals, more individuals with high educational attainment, more middle-income-level individuals, and fewer individuals who identified as religious. Of all respondents with complete data, 26 reported SG scores that were all equal (i.e., were invariant), and 50 reported SG scores that were illogical, for a total of 76 who were excluded from the subset analysis. Respondents included in the subset sample were slightly younger, more educated, less religious, and more often white than those in the entire survey sample (Table 1).

**Table 1:** Sample characteristics and US population comparison

	All survey respondents n = 368	Logical, variant subset n = 292	US population 2000-2008 estimates
	No.(%)	No.(%)	% (source)
Age, years (mean, sd)	39.5(14.7)	38.0(14.5)	36.6[22]
Female	279(76%)^1^	222(76%)	50.7%[23]
Race			
White	322(88%)	263(90%)	79.8%[23]
Black/African American	23(6%)	13(4%)	12.8%
Asian	8(2%)	8(3%)	4.5%
Other races/multiracial	15(4%)	8(3%)	2.9%
Education			
High school or less	11(3%)^1^	2(1%)	45.2%[23]
Some college	91(25%)	66(23%)	27.9%
4-year college graduate	93(25%)	81(28%)	17.8%
More than college	173(47%)	142(49%)	9.1%
Annual household income			
<$25,000	52(14%)^1^	38(13%)	24.8%[24]
≥$25,000 and <$50,000	113(31%)	92(32%)	24.9%
≥$50,000 and <$100,000	121(34%)	96(34%)	29.9%
≥$100,000	73(21%)	59(21%)	20.5%
Children < 18 years in household	123 (33%)	86 (29%)	50%[25]
Religious (yes)	205(56%)^1^	151(52%)	85%[26]
Health status			
Excellent	80(22%)	68(23%)	35%[27]
Very good	172(47%)	138(47%)	30%
Good	97(26%)	73(25%)	24%
Fair	19(5%)	13(4%)	7%
Poor	0	0	2%

No = number; sd = standard deviation

Percentages may not sum to 100 due to rounding

^1 ^Missing items from respondents: 1 respondent skipped gender question, 1 skipped education question, 2 skipped religion question, and 9 skipped income question

Mean standard gamble scores for the health states decreased as the severity of the states increased, in both the entire sample and the subsample (Table 2). Mean scores for the mild state ranged from 0.84-0.86 for the complete sample and the subsample, 0.68-0.67 for the moderate state, and 0.45-0.38 for the severe state, respectively. In adjusted analyses, SG scores were not significantly affected by the added description of time of onset to the health state scenario compared with omission of this information, with the exception of the mild health state in the logical/variant subsample (Table 2). For this state, SG scores were slightly lower for those respondents for whom the state was described as beginning 6 months prior ("prior onset") compared with respondents who were given no indication of the time of onset (regression coefficient = -0.07, 95% CI = [-0.13, -0.01]). For all states and samples, there was no significant difference between states described as prior onset compared with those described as current onset (results not shown). Age was the only respondent characteristic that had a consistently significant association with SG scores, with increased age associated with lower scores across health state severity and sample. The presence of dependent children in the household was associated with higher scores for the mild health state in both samples (Table 2).

**Table 2:** Generalized linear model predicting standard gamble scores by health state severity, all respondents and subset meeting logic and variance criteria: regression coefficients and 95% confidence intervals

	Mildly severe state	Moderately severe state	Severe state
Variable	estimate (95% CI)	estimate (95% CI)	estimate (95% CI)
**All respondents (n = 368; current onset n = 122, prior onset n = 117, unspecified onset n = 129)**			

	Mean(sd) = 0.84(0.25)	Mean(sd) = 0.68(0.32)	Mean(sd) = 0.45(0.37)

Time of onset*:			
Prior	-0.05 (-0.12, 0.01)	-0.07 (-0.15, 0.01)	-0.09 (-0.18, 0.01)
Current	-0.01 (-0.08, 0.05)	-0.03 (-0.11, 0.05)	-0.05 (-0.14, 0.04)
Health status	0.01 (-0.02, 0.05)	0.00 (-0.05, 0.04)	-0.03 (-0.08, 0.01)
Age (years)	**-0.003 (-0.005, -0.001)**	**-0.004 (-0.006, -0.001)**	-0.002 (-0.005, 0.001)
White race	0.01 (-0.07, 0.09)	-0.04 (-0.14, 0.06)	**-0.15 (-0.26, -0.03)**
Female	0.02 (-0.05, 0.08)	0.00 (-0.04, 0.10)	0.01 (-0.08, 0.10)
Dependent children	**0.11 (0.05, 0.18)**	0.06 (-0.03, 0.14)	0.03 (-0.06, 0.13)
College educated	0.04 (-0.02, 0.10)	-0.03 (-0.11, 0.04)	-0.05 (-0.03, 0.04)
Religious	0.0 (-0.05, 0.05)	0.03 (-0.04, 0.10)	0.07 (-0.01, 0.14)

**Logical, variant subset (n = 292; current onset n = 100, prior onset n = 93, unspecified onset n = 99)**			

	Mean(sd) = 0.86(0.21)	Mean(sd) = 0.67(0.30)	Mean(sd) = 0.38(0.33)

Time of onset*:			
Prior	**-0.07 (-0.13, -0.01)**	-0.04 (-0.13, 0.04)	-0.07 (-0.17, 0.02)
Current	-0.02 (-0.08, 0.04)	-0.00 (-0.09, 0.08)	-0.04 (-0.14, 0.05)
Health status	0.01 (-0.02, 0.04)	-0.01 (-0.05, 0.04)	**-0.06 (-0.10, -0.01)**
Age (years)	**-0.002 (-0.004, -0.000)**	**-0.004 (-0.007, -0.002)**	**-0.006 (-0.009, -0.003)**
White race	0.03 (-0.05, 0.12)	-0.05 (-0.17, 0.07)	-0.04 (-0.17, 0.08)
Female	0.00 (-0.06, 0.06)	0.01 (-0.08, 0.09)	-0.03 (-0.12, 0.06)
Dependent children	**0.08 (0.01, 0.15)**	0.04 (-0.05, 0.14)	0.05 (-0.05, 0.15)
College educated	-0.03 (-0.10, 0.03)	-0.04 (-0.13, 0.05)	-0.03 (-0.13, 0.06)
Religious	-0.01 (-0.06, 0.04)	0.03 (-0.04, 0.10)	0.02 (-0.06, 0.09)

* No time of onset specified ("unspecified onset") is reference

CI = confidence interval; sd = standard deviation

**Bold **= significant at p ≤ 0.05

## Discussion

Utility measurement is a fundamentally complex task, both for investigators designing tools and respondents providing values[16]. In the context of eliciting community-perspective preferences for hypothetical health states, the way in which a health state is described can have substantial impact on how a state is valued[17], as can the valuation technique used[8]. This research explored one specific element of the health state description for the valuation of hypothetical states, how the timing of the health state's occurrence is described, and specifically, whether the time of onset is included in the description and whether that onset was recent. This question addresses the known distinction between patient and community-perspective values for the same health state by attempting to decipher the inferred meaning of omitted health state description information in community-perspective valuations. Time of onset of a condition may infer adaptation to disease, the transition between healthy and ill, and affective states such as hopeless and despair associated with long-term conditions. These elements may contribute to the observed difference in values between patient and community perspective values, and hence the inclusion of this information in hypothetical health state descriptions may increase understanding of the condition for individuals lacking experience with it. While exploratory, this research found that the inclusion of this detail in health state descriptions did not have a measureable effect on the values provided, even when excluding utility survey responses that demonstrate elements of misunderstanding or miscomprehension, a procedure likely to improve the validity of results. We speculate that the common practice of omitting time of onset in descriptions of health state scenarios for the elicitation of community-perspective utilities may not induce bias into results, either because such information is not salient to community values or that the inferred information used by respondents is already accurate. In either case, we cannot provide evidence from this study in favor of inclusion or exclusion and suggest further exploration of these preference elements.

Our results suggest a number of hypotheses about the community-perspective utility elicitation process that may be useful for preference assessment methods. First, it may be that time of onset is not salient to community-perspective survey respondents when faced with a utility survey of average complexity. Survey elements or formats specifically designed to focus attention or consideration on onset were intentionally omitted from this survey to mimic conventional survey design. Attention may have to be drawn specifically to time of onset for respondents to consider this in valuations. Further research could explore whether increased attention alters values.

Second, community members may recognize differences in onset, but may not be able to forecast differences in valuation depending on experience with a state or adaptation, and hence may genuinely value states of different onset similarly[18,19]. There is contradictory evidence in the literature regarding the relative value of states of different onset, but supportive of respondents' ability to distinguish across timing and to assign value. Damschroeder and others compared "pre-existing" and "new onset" conditions and found the "new onset" conditions were valued lower (i.e., worse) in person trade-offs[5]. These comparative results imply that survey respondents may anticipate adaptation to disease that occurs with pre-existing conditions, or may otherwise believe that newly-occurring conditions are worse than those that have existed over time. On the other hand, Lieu and others found evidence that recent onset conditions were inferred as temporary and thus possibly better (i.e., less negative) than those that are permanent[20]. Some of our data support the hypothesis that long-term conditions are worse to endure rather than better, as indicated by the negative premium placed on prior onset for mild conditions in our subset analysis. This finding runs counter to the prevailing notion of adaptation to disease that is observed among patient-perspective valuations. Anecdotal evidence from commentary provided in our survey suggested that some respondents associated prior onset with increased hopelessness and dread, and therefore assigned lower utilities to pre-existing conditions. In sum, while patient-perspective utilities generally demonstrate adaptation to disease, community-perspective values show more varied response to the inclusion of health state descriptors that approximate longer-term conditions, such as prior onset and pre-existing conditions, and it is not yet clear whether adaptation can or is incorporated into community-perspective values elicited using hypothetical health state descriptions.

An alternative explanation for a difference in values due to time of onset is that the actual transition between healthy and ill represents an immediate loss in health that individuals value disproportionately negatively, as posited by prospect theory[21]. This hypothesis would be supported by lower scores for current compared with prior onset conditions, which was not seen in our data but was supported by Damschroeder's findings[5]. The literature confirms that time of onset has an effect on values among some community-perspective respondents using some measurement techniques, so is clearly an important element of the elicitation task. Our results add to this debate but do not offer conclusive evidence for or against the inclusion of time of onset in descriptions. Further research into the cognitive mechanisms underlying the distinctions in processing or assessment of health state descriptions may illuminate the optimal elements to be included in health state descriptions.

Though suggestive of areas for further research and hypotheses, our results should of course be considered exploratory in nature due to acknowledged limitations in our design and sample. We attempted to mimic typical utility survey design in question framing, and to provide decision-support through warm-up questions, opportunities to revise answers and visual aids, but in doing so did not specifically draw respondents' attention to the time of onset element of the descriptions. Our intent was to study utility elicitation as it is currently conducted, and provide insight into the conventional process. Our approach may have sacrificed measurement precision for practical applicability. Moreover, we used internet administration for our survey because of its convenience and the increasing reliance on this mode in the utility measurement field. Internet format allows respondents to proceed at their desired pace through the survey, but as a self-administered format, may permit inattention to details compared with in-person administration. And finally, our sample was selected of convenience, and while typical of internet survey samples, was substantially different from the US population on factors that affect preferences and utility responses (such as education). We do not know whether the observed sample differences are relevant to how individuals consider onset of disease in preferences, or whether other, unobserved differences with our sample relative to the US population have biased our results. Our results should be considered as informative for survey design rather than definitive regarding the inclusion of onset information in health state description.

## Conclusion

In conclusion, the goal of this paper was to motivate additional exploration of how community-perspective respondents assign value to transitioning into a health state versus living in it over time, and how timing of health states' occurrence are reflected in values for hypothetical health state descriptions. These elements of disease are important to patients' decision making but may be overlooked by traditional community-perspective utility elicitation techniques that ignore onset, and by implication the transition between states. Perfecting our methods of community-perspective preference assessment will provide a stronger and more valid basis for evaluations that depend on these inputs, and lead to improved analyses and hence decision making.

## Competing interests

The authors declare that they have no competing interests.
